# Investigation of Gal-3 Expression Pattern in Serum and Cerebrospinal Fluid of Patients Suffering From Neurodegenerative Disorders

**DOI:** 10.3389/fnins.2018.00430

**Published:** 2018-06-29

**Authors:** Ghulam M. Ashraf, Saleh S. Baeesa

**Affiliations:** ^1^King Fahd Medical Research Center, King Abdulaziz University, Jeddah, Saudi Arabia; ^2^Division of Neurosurgery, College of Medicine, King Abdulaziz University, Jeddah, Saudi Arabia

**Keywords:** Galectin-3, serum, cerebrospinal fluid, Alzheimer’s disease, amyotrophic lateral sclerosis

## Abstract

We performed this study to investigate the possibility of a definitive pattern of Galectin-3 (Gal-3) expression in the cerebrospinal fluid (CSF) and serum of Alzheimer’s disease (AD) and Amyotrophic Lateral Sclerosis (ALS) patients. In our study, we collected the CSF and serum samples of 31 AD patients, 19 ALS patients and 50 normal healthy subjects (controls). Quantitative ELISA measured Gal-3 concentrations in CSF and serum samples. A comparative analysis was performed to analyze and understand the Gal-3 expression pattern. A number of neuropsychological assessments and statistical analyses were carried out to validate our findings. Recent researches have established the role of galectins in various neurodegenerative disorders (NDDs), but a definitive pattern of galectin expression is still elusive. A significant difference was observed in serum and CSF Gal-3 concentrations between AD patients and healthy controls. The difference in serum and CSF Gal-3 concentrations between ALS patients vs. controls was lesser as compared to AD patients vs. controls. The difference in serum and CSF Gal-3 concentrations of AD vs. ALS patients was not significant. The MMSE score and serum and CSF Gal-3 concentrations in AD and ALS patients, and controls exhibited a significant positive correlation. The findings of the present study are expected to provide an insight into the definitive pattern of Gal-3 expression in AD and ALS patients, and might thus establish Gal-3 as a strong biomarker. This in turn will open up new and promising research avenues targeting the expression of galectins to modulate the progression of NDDs, and pave the way for novel therapeutic options.

## Introduction

Galectins are mammalian class of an otherwise large family, lectin, and characterized as glycoproteins, which have been reported to be expressed in almost all vital organs and involved in almost all significant biological functions (Hasan et al., 2007; Viguier et al., 2014). Gal-3 has been reported to exhibit an altered pattern of expression in patients suffering from NDDs like AD, ALS, PD, and VaD. While the exact molecular mechanisms behind the pathogenesis of various NDDs remain elusive, the experimental findings from the samples obtained from the patients continue to generate remarkable inputs related to possible pathogenetic channels of these disorders. Gal-3 is present in intracellular (cytosol and nucleus) as well as extracellular spaces, and has been reported to be expressed by almost all cell types (Hasan et al., 2007; Fortuna-Costa et al., 2014). Gal-3 being a multifunctional protein has been reported to be involved in plethora of physiological functions like immune activation, apoptosis, angiogenesis and fibrosis, which in turn have been intricately associated with the development and progression of various NDDs (Ashraf et al., 2010b, 2011; Radosavljevic et al., 2012; Tam and Pasternak, 2012). Gal-3 has also been reported to inhibit the peripheral nerve generation post axotomy (Narciso et al., 2009; DeFrancesco-Lisowitz et al., 2015), act as cell surface receptor for AGEs (Yamamoto and Yamamoto, 2012; Chen H. et al., 2017), and exacerbate the CNS damage in experimental autoimmune encephalomyelitis (EAE) (Jiang et al., 2009; de Oliveira et al., 2015). Microglial Gal-3 has significant role in neuroinflammation process in chronic ALS and TBI (Lerman et al., 2012; Boza-Serrano et al., 2014; Yip et al., 2017). A detrimental role of Gal-3 has been established in inflammation associated with CNS related prion infections (Mok et al., 2007). Gal-3 elevation induced by microglia during the progression of Lewy body dementia and ALS can act as effective indicator of neurodegenerative immune response (Zhou et al., 2010; Surendranathan et al., 2015; Garden and Campbell, 2016). Moreover, onset of remyelination has been favored by microglial Gal-3, either by directly effecting the differentiation of oligodendrocytes or through M2 cell polarization (Cherry et al., 2014; Franco et al., 2015; Rinaldi et al., 2016). Elevated levels of Gal-3 have been observed in the CSF of patients with TBI, ALS and newborn infants after birth asphyxia (Wuolikainen et al., 2011; Sävman et al., 2013; Yip et al., 2017). Gal-3 deregulates hippocampus-dependent memory formation via intracellular as well as extracellular mechanisms (Chen Y.C. et al., 2017). Proteomics approaches has established Gal-3 as a candidate biomarker for ALS (Zhou et al., 2010). Gal-3 has also been reported to be involved in AD neuropathogenesis, and could be a potential biomarker for AD (Wang et al., 2015). Moreover, Gal-3 has been identified in central as well as peripheral nervous system in Schwann cells, endothelial cells, microglia/macrophages, and astrocytes; and the activation of endothelial cells and microglia has been intricately associated with AD pathogenesis (Zlokovic, 2008; Ottum et al., 2015). These studies proclaim elevated Gal-3 expression pattern in AD and ALS patients.

The idea of the proposed research study cropped from the above-mentioned reports suggesting altered expression level of Gal-3 in various NDDs. The detrimental role of Gal-3 in diseases like ALS and AD suggest that lysosomal dysfunctions combined with reduced autophagy can further stimulate neurodegeneration. Elevated levels of Gal-3 in serum and CSF might induce enhanced inflammation, apoptosis and neurodegeneration. There are few individual studies on either serum or CSF, which suggest Gal-3 as possible biomarker of NDDs. The findings of the present study suggesting a definitive Gal-3 expression pattern in serum as well as CSF samples of AD and ALS patients will firmly affirm the role of Gal-3 as potential biomarker for these diseases.

## Materials and Methods

### Ethics Statement

This study was carried out in accordance with the recommendations of National Institute of Neurological and Communicative Disorders and Stroke AD and Related Disorders Association and International Classification of Diseases, Tenth Revision, Diagnostic and Statistical Manual of Mental Disorders (Third Edition) (McKhann et al., 1984). The KAUH (King Abdulaziz University Hospital) ethical committee approved the protocol. All subjects gave written informed consent in accordance with the Declaration of Helsinki.

### Patients and Neuropsychological Assessment

The AD (31) and ALS (19) patients as well as healthy individuals (50) serving as controls were recruited from Division of Neurosurgery, College of Medicine, King Abdulaziz University, Jeddah, Saudi Arabia under the careful supervision of Prof. Saleh Baeesa. Each participant was requested to provide written informed consent. The clinical severity of cognitive status was assessed by the standard MMSE (Mini Mental Status Examination) (Folstein et al., 1975). All the neuropsychological assessments were carried out by trained MD (Neurosurgery) students under the supervision of Prof. Saleh Baeesa, and included tests like SKT (Lehfeld and Erzigkeit, 1997; Flaks et al., 2006), TMT (Tombaugh, 2004), and FOME (Fuld, 1980). The cut off scores of all neuropsychological tests were adjusted for educational level and age. The participants were assessed with 21-item Hamilton depressive scale and euthymia was defined as a score of less than 8 (Hamilton, 1960). To exclude metabolic and vascular etiologies, magnetic resonance imaging (MRI) studies and blood tests (blood chemistry, blood lipid profile, complete blood count, folic acid, thyroid function, vitamin B12 dosage and syphilis test) were carried out. A consensus was developed before the clinical diagnoses by taking into account all the laboratory and clinical informations.

### Sample Collection

CSF as well as serum samples were collected from 31 patients with AD and 19 patients with ALS. The collected samples were frozen at −80°C until assayed. Samples were also collected from 50 healthy elderly individuals, which served as controls. For CSF sampling, lumbar puncture (LB) using 20G spinal needle was performed using a 20 Gauge, 3.5 inch Quincke point spinal needle (Becton, Dickinson and Company) under local anesthesia in recumbent position at the L3/L4 spine interspace or below of each subject. Every CSF sample collected was approximately 5–8 ml in volume. The CSF samples were collected in Falcon polypropylene tubes (BD Biosciences, Franklin Lakes, NJ, United States). The serum as well as CSF samples were collected early in the morning between 8 and 9 am. The collected CSF samples were centrifuged at 2500 rpm for 5 min to exclude blood contaminants. The samples were then aliquoted in smaller concentrations of 250 μl per aliquot and frozen instantly at −80°C until used for further analyses. While performing the experiments, we freeze thawed only the required number of aliquots as per the requirements of sample concentration needed for the experiment. All the patients and control individuals were fasting since the evening before at the time of LP, and were requested to abstain from smoking. Most of the CSF and serum samples were analyzed within 8 weeks of their collection. This duration was mainly attributed to the fact that before opening an ELISA kit, we made sure that we have collected at least 20 cumulative samples of CSF and serum.

### Inclusion/Exclusion Criteria

- All the AD and ALS patients included in this study were diagnosed with the disease since at least 2 years.
- Only those patients and controls were included who gave their consent for the study either by themselves or with the help of their close kin.
- All the subjects under the prescription of NSAIDs or any other drugs interfering with the neuroinflammatory system at the time of sample collection were excluded.
- All the subjects reported for chain smoking were excluded; the information was confirmed by accompanying kin.

### Limitations

- An important limitation of our study is relatively small sample size as compared with those used by large-scale multicentric studies.
- We collected the serum and CSF samples in a span of 2 years from almost all the NDDs patients like AD, PD, ALS, MCI, VaD, and MS based on their consent.
- For this study, we only selected the two group of patients with maximum number of samples, and those turned out to be AD (31 patients) and ALS (19 patients). The PD (7 patients), MCI (9 patients), VaD (7 patients) and MS (4 patients) were much lesser in number, and we chose to not include them in this study and wait for more samples to design an effective analyses.

### Measurement of Gal-3 Concentrations in Serum, CSF and Control Samples

The concentrations of Gal-3 in serum, CSF and control samples were measured by Abcam’s Galectin-3 *in vitro* SimpleStep ELISA^TM^ kit (ab188394). Briefly, 50 μL of all samples was added to respective wells of polystyrene 96-well plates which were pre-coated with antibodies against Gal-3 (i.e., anti-Gal-3-antibodies), followed by the addition of 50 μL antibody cocktail to each and every well. The treated plate was then sealed followed by incubation at RT (room temperature) for 1 h, and finally shaked at 400 rpm on a plate shaker at RT for 2 h. The plate was then washed three times with 350 μL wash buffer, the plate was inverted and blotted against clean paper towels to remove excess liquid. This was followed by the addition of 100 μL TMB substrate to every well, and incubation at 400 rpm on a plate shaker for 10 min in dark. In the last step, each well was added with 100 μL stop solution, and the plate was shaken for 1 min to mix the solutions. The endpoint reading was recorded at 450 nm in ELISA reader (ELx50, Biotek).

### Data Interpretation and Statistical Analysis

The differences in cognitive performance, sociodemographic characteristics, and Gal-3 concentrations were analyzed by ANOVA test, with LSD tests for pairwise *post hoc* test between groups. Chi square tests were used to analyze the differences in gender distribution. The differences in scores of baseline and follow up neuropsychological tests for AD and ALS groups were analyzed by LSD test. ROC curves were built to determine the sensitivity (SE), specificity (SP), and area under the curve of Gal-3 biomarker for the discrimination between AD and controls, and ALS and controls.

## Results and Discussion

In relation to the present study, Gal-3 has been reported to be involved in the physiology of almost all types of immune cells (Rabinovich et al., 2012; Chung et al., 2013; Asayama et al., 2017). The information on the Gal-3 expression regulation, however, is quite limited and needs further elucidation. In the present study, we investigated the expression pattern of Gal-3 levels in serum and CSF of AD and ALS patients, and tried to explore the potential associations with the clinical symptoms of these disorders.

The AD patients were on average older than ALS patients were, and thus scored lower on MMSE test. No significant difference was found in years of education and gender distribution between AD, ALS and controls groups. The baseline characteristics and demographic parameters have been summarized in **Table 1**. The results of two consecutive neuropsychological evaluations performed at an interval of at least 1 year have been summarized in **Table 2**. In the ALS group, the scores in all neuropsychological tests remained stable over time, whereas in the AD group, there was a decline in TMT-A, FOME, and SKT scores in the same period.

**Table 1:** Demographic parameters and baseline characteristics.

Characteristics	AD	ALS	Control	*P*		
	(*n* = 31)	(*n* = 19)	(*n* = 50)	(AD vs. controls)	(ALS vs. controls)	(AD vs. ALS)
Age (years)	66.8 ± 7.8	64.1 ± 6.9	74.9 ± 7.3	0.06	0.07	0.03
Years of education	9.16 ± 5.67	10.65 ± 5.14	13.34 ± 6.12	0.03	0.04	0.38
Males	18	11	29	0.03	0.04	0.04
Females	13	8	21			
MMSE score	20.7 ± 5.2	23.4 ± 6.2	29.7 ± 0.8	<0.001	<0.001	<0.001
Serum Gal-3 (ng/mL)	11.19 ± 3.67	10.45 ± 3.48	8.76 ± 3.03	0.02	0.02	0.07
CSF Gal-3 (ng/mL)	8.37 ± 2.79	5.19 ± 2.23	7.92 ± 2.56	0.02	0.02	0.07

*AD, Alzheimer’s disease; ALS, amyotrophic lateral sclerosis; MMSE, Mini-Mental State Examination.*

**Table 2:** Neuropsychological performances of AD and ALS patients.

Test	ALS			AD		
	Baseline	Follow-up	*P*	Baseline	Follow-up	*P*
FOME	24.21 ± 6.26	24.19 ± 6.81	0.54	26.45 ± 5.84	21.2 ± 5.94	0.01
MMSE	21.86 ± 4.23	22.02 ± 4.16	0.62	24.84 ± 3.27	23.1 ± 3.96	0.65
TMT-A	68.2 ± 28.34	67.95 ± 24.2	0.12	70.55 ± 38.48	93.21 ± 41.22	0.05
TMT-B	144.28 ± 50.25	146.9 ± 54.23	0.51	159.75 ± 55.34	215 ± 88.65	0.06
SKT	2.95 ± 3.28	3.24 ± 4.19	0.43	5.84 ± 4.55	8.76 ± 5.18	0.02

*AD, Alzheimer’s disease; ALS, amyotrophic lateral sclerosis; FOME, Fuld object memory evaluation; MMSE, mini-mental state examination; TMT-A, trail making test A; TMT-B, trail making test B; SKT, short cognitive test.*

**FIGURE 1:**
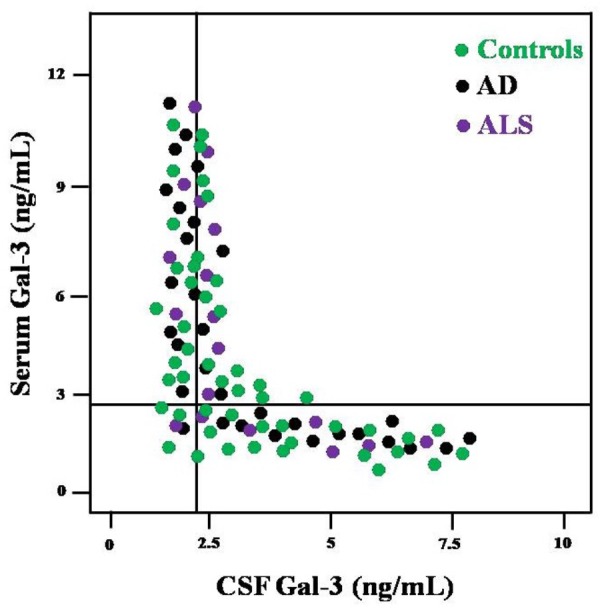
Distribution of AD, ALS and control subjects according to the values of CSF Gal-3 (*x*-axis) and serum Gal-3 (*y*-axis).

Based on SE and SP values, ROC curves were drawn to determine the cutoff scores for serum as well as CSF Gal-3 concentrations (**Table 3**). Serum Gal-3 concentrations >8.32 ng/mL (SE = 87% and SP = 74%) and CSF Gal-3 concentrations >7.64 (SE = 83% and SP = 69%) best differentiated the AD, ALS and control groups. We also investigated whether the Gal-3 concentrations might alter the diagnostic accuracy. **Figure 1** represents the distribution of subjects according to the serum and CSF Gal-3 concentrations in AD, ALS and control groups. Our findings suggested that the AD patients had a distribution almost similar those of ALS patients.

**Table 3:** Cutoff scores, sensitivity and specificity values of serum and CSF Gal-3 concentrations differentiating the AD patients from controls, ALS patients from controls, and AD patients and ALS patients.

	Cutoff	AD (*n* = 31) vs. controls (*n* = 50)		ALS (*n* = 19) vs. controls (*n* = 50)		AD (*n* = 31) vs. ALS (*n* = 19)	
		Sensitivity (%)	Specificity (%)	Sensitivity (%)	Specificity (%)	Sensitivity (%)	Specificity (%)
Serum Gal-3 (ng/mL)	>8.32	87	74	82	71	88	76
CSF Gal-3 (ng/mL)	>7.64	83	69	78	65	85	73

**FIGURE 2:**
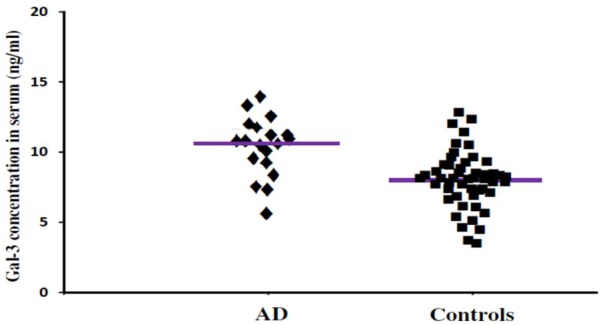
The concentrations of Gal-3 in serum (ng/mL) of AD patients and healthy controls. AD patients showed enhanced Gal-3 concentrations in serum as compared to the healthy controls (AD vs. healthy controls [mean ± SD] 11.19 ± 3.67 vs. 8.76 ± 3.03 ng/mL; *P* = 0.022).

**FIGURE 3:**
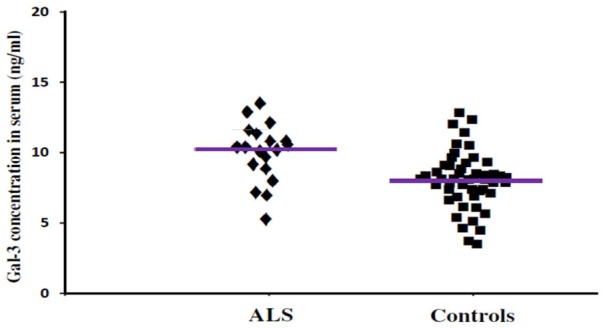
The concentrations of Gal-3 in serum (ng/mL) of ALS patients and healthy controls. ALS patients showed enhanced Gal-3 concentrations in serum as compared to the healthy controls (ALS vs. healthy controls [mean ± SD] 10.45 ± 3.48 vs. 8.76 ± 3.03 ng/mL; *P* = 0.019).

**FIGURE 4:**
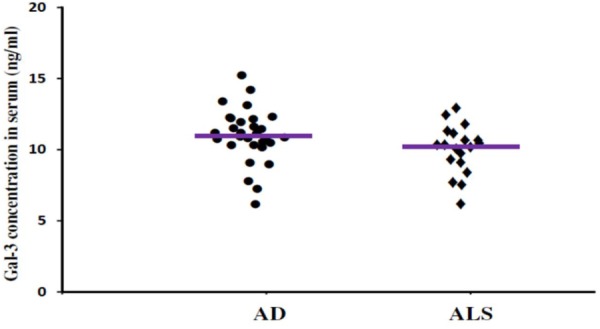
The concentrations of Gal-3 in serum (ng/mL) of AD and ALS. There was no significant difference in the concentrations of Gal-3 in serum of AD and ALS patients (AD vs. ALS [mean ± SD] 11.19 ± 3.67 vs. 10.45 ± 3.48 ng/mL; *P* = 0.719).

**FIGURE 5:**
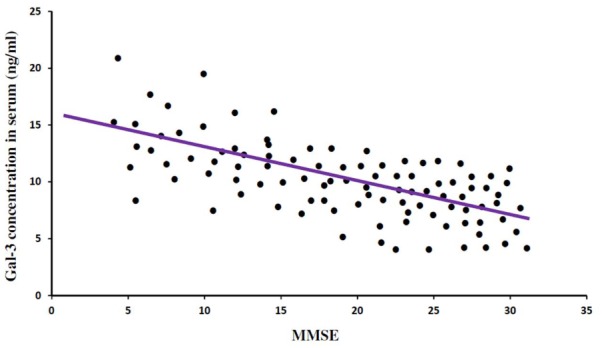
Mini Mental Status Examination (MMSE) and serum Gal-3 concentrations. A significant correlation was observed between MMSE score as a measure of cognitive status and serum Gal-3 levels (*P* = 0.306; *P* < 0.001) in all the patients (AD = 31, ALS = 19, Controls = 50).

In order to incorporate the difference in age as a factor between AD and ALS patients, and controls; age was used as ANOVA covariate. Visible differences were observed in serum Gal-3 concentrations between the three different groups (AD, ALS, and healthy controls) (*P* = 0.005). Remarkable difference in the serum Gal-3 concentrations was observed between AD and ALS patients during the LSD test. **Figure 2** depicts that the difference in the serum Gal-3 concentrations between AD patients and healthy controls was significant [AD vs. controls (mean ± SD) 11.19 ± 3.67 vs. 8.76 ± 3.03 ng/mL; *P* = 0.02]. Similarly, **Figure 3** depicts that the difference in serum Gal-3 concentrations between ALS patients and controls was significant [ALS vs. controls (mean ± SD) 10.45 ± 3.48 vs. 8.76 ± 3.03 ng/mL; *P* = 0.02], but the difference was lesser as compared to AD vs. controls. **Figure 4** depicts that the difference in serum Gal-3 concentrations of AD and ALS patients was not significant [AD vs. ALS (mean ± SD) 11.19 ± 3.67 vs. 10.45 ± 3.48 ng/mL; *P* = 0.07]. **Figure 5** depicts that the MMSE score and serum Gal-3 concentrations in AD and ALS patients, and controls exhibited a significant positive correlation (*P* = 0.306; *P* < 0.001).

**FIGURE 6:**
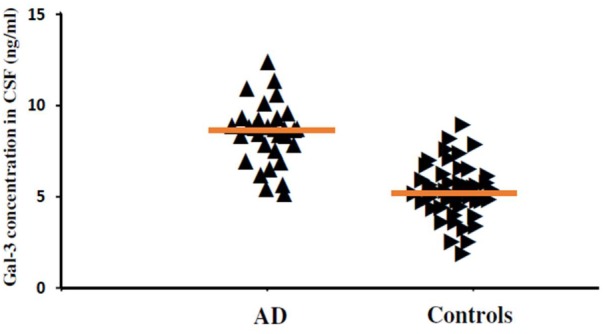
The concentrations of Gal-3 in CSF (ng/mL) of AD patients and healthy controls. AD patients showed enhanced Gal-3 concentrations in CSF as compared to the healthy controls (AD vs. healthy controls [mean ± SD] 8.37 ± 2.79 vs. 5.19 ± 2.23 ng/mL; *P* = 0.019).

**FIGURE 7:**
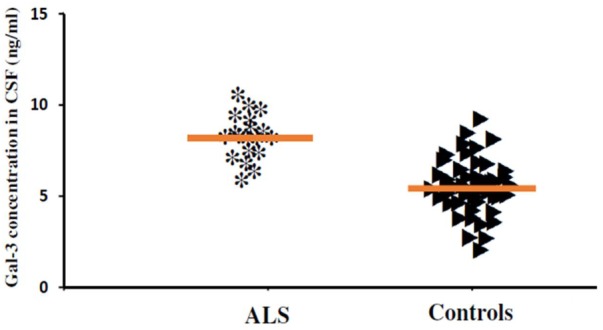
The concentrations of Gal-3 in CSF (ng/mL) of ALS patients and healthy controls. ALS patients showed enhanced Gal-3 concentrations in CSF as compared to the healthy controls (ALS vs. healthy controls [mean ± SD] 7.92 ± 2.56 vs. 5.19 ± 2.23 ng/mL; *P* = 0.017).

**FIGURE 8:**
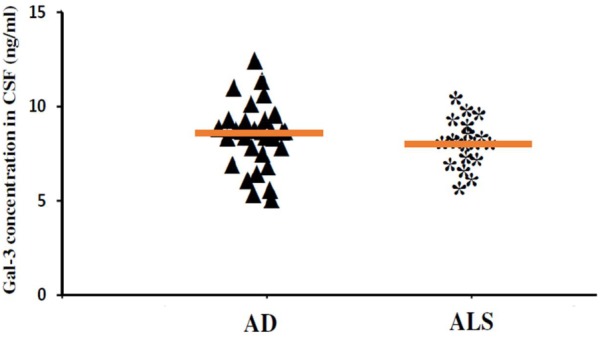
The concentrations of Gal-3 in CSF (ng/mL) of AD and ALS. There was no significant difference in the concentrations of Gal-3 in CSF of AD and ALS patients (AD vs. ALS [mean ± SD] 8.37 ± 2.79 vs. 7.92 ± 2.56 ng/mL; *P* = 0.734).

**FIGURE 9:**
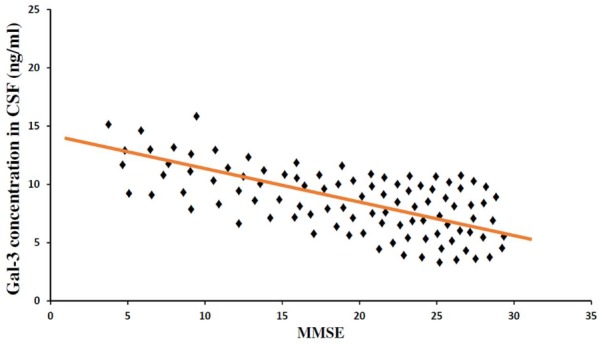
Mini Mental Status Examination and CSF Gal-3 concentrations. A significant correlation was observed between MMSE score as a measure of cognitive status and CSF Gal-3 levels (*P* = 0.306; *P* < 0.001) in all the patients (AD = 31, ALS = 19, Controls = 50).

In order to incorporate the difference in age as a factor between AD and ALS patients, and controls; age was again used as ANOVA covariate. Visible differences were observed in CSF Gal-3 concentrations between the three different groups (AD, ALS, and healthy controls) (*P* = 0.005). Remarkable difference in the CSF Gal-3 concentrations was observed between AD and ALS patients during the LSD test. **Figure 6** depicts that the difference in the CSF Gal-3 concentrations between AD patients and healthy controls was significant [AD vs. healthy controls (mean ± SD) 8.37 ± 2.79 vs. 5.19 ± 2.23 ng/mL; *P* = 0.02]. Similarly, **Figure 7** depicts that the difference in CSF Gal-3 concentrations between ALS patients and controls was significant [ALS vs. healthy controls (mean ± SD) 7.92 ± 2.56 vs. 5.19 ± 2.23 ng/mL; *P* = 0.02], but the difference was lesser as compared to AD vs. controls. **Figure 8** depicts that the difference in CSF Gal-3 concentrations of AD and ALS patients was not significant [AD vs. ALS (mean ± SD) 8.37 ± 2.79 vs. 7.92 ± 2.56 ng/mL; *P* = 0.07]. **Figure 9** depicts that the MMSE score and CSF Gal-3 concentrations in AD and ALS patients, and controls exhibited a significant positive correlation (*P* = 0.291; *P* < 0.001).

The findings of our study demonstrated that the expression pattern of Gal-3 was significantly enhanced as compared to the controls in serum (**Figures 2, 3**) as well as CSF samples (**Figures 6, 7**) of AD and ALS patients. This enhanced expression of Gal-3 can be attributed to the activation and regulation of immune system in AD and ALS patients (Filer et al., 2009; Bauer et al., 2016; Yip et al., 2017). However, the difference in Gal-3 expression in serum (**Figure 4**) as well as CSF samples (**Figure 8**) between AD and ALS patients was not significant. This finding seems evident as the Gal-3 expression is altered to a considerable degree in AD as well as ALS patients, in reference to the control samples. This finding also suggests that Gal-3 is an aspecific marker that will discriminate every neurodegenerative/neuroinflammatory-related condition as opposed to a control population, and lacks specificity to discriminate between various dementias. A significant positive correlation between serum and CSF Gal-3 levels and MMSE score was observed (**Figures 5, 9** and **Table 1**). The scores of neuropsychological tests remained stable in ALS patients over time, whereas in the AD group, there was a decline in TMT-A, FOME, and SKT scores in the same period (**Table 2**). This finding is well received, as there is hardly any memory loss is ALS patients, whereas there is a rapid loss of memory and cognitive skills in AD patients.

To our knowledge, the findings that serum and CSF Gal-3 levels are elevated in AD as well as ALS patients are one of the first of its kind to be reported from Saudi Arabia. Gal-3 functional roles have been reported in CNS (Bresalier et al., 1997; Reichert and Rotshenker, 1999). Gal-3 inductions are evident in various pathological processes of brain diseases such as prion diseases, ALS, AD, ischemic brain lesions, and PD (Jin et al., 2007; Lerman et al., 2012; Ashraf et al., 2015). The possible physiological role of Gal-3 in AD and ALS can be investigated by exploring its already established biological functions like their role in inflammation and apoptosis (extracellular Gal-3 being proapoptotic, and intracellular Gal-3 being anti-apoptotic) (Yu et al., 2002; Nangia-Makker et al., 2007; Henderson and Sethi, 2009; Ashraf et al., 2010a; Siwicki et al., 2016). The activation of Gal-3 dependent TLR4 induces a sustained microglia activation, which in turn prolongs the inflammatory response in the brain (Burguillos et al., 2015). Gal-3 generates an enhanced inflammatory response by suppressing the production of interleukin 10 (IL-10), which is anti-inflammatory neuroprotective cytokine released by microglia during the progression of AD (Zhou et al., 2010; Di Bona et al., 2012). In a recent study, Gal-3 levels in plasma were reported to be highly enhanced in ALS patients with limb onset of the disease (Yan et al., 2016). This effect was especially more pronounced in female ALS patients and was found to be positively correlated with disease duration. In another study, Gal-3 deletion exacerbated microglial activation and accelerated the disease progression and demise in SOD1(G93A) mouse model of ALS (Lerman et al., 2012). These findings suggest Gal-3 as a crucial factor associated with ALS.

Based on above discussions, the elevated levels of Gal-3 in serum and CSF samples of AD and ALS patients investigated in our study potentially suggests activation of inflammation and apoptosis, and impairment of neurodegenerative events in these patients. Our study successfully demonstrated a definitive association between the serum and CSF levels of Gal-3 and the cognitive status in AD and ALS patients and healthy individuals (controls). The secretory nature of Gal-3 and its detectable level in serum and CSF strongly proclaim this dynamic molecule as potential biomarker for AD and ALS in particular, and other NDDs in general. Our interesting findings suggest the need to explore and investigate the role of other galectins (Gal-1 to Gal-15) in various NDDs. These crucial findings strongly entail the need of future evaluation in prospective patient’s cohort, which in turn can put galectins, especially Gal-3, in clinical trials.

## Author Contributions

GA conceived the study, performed all the experiments, analyzed the data, and compiled the whole manuscript. SB provided the CSF and serum samples.

## Conflict of Interest Statement

The authors declare that the research was conducted in the absence of any commercial or financial relationships that could be construed as a potential conflict of interest.

## Abbreviations

AD: Alzheimer’s disease
AGEs: advanced glycation end products
ALS: amyotrophic lateral sclerosis
ANOVA: analysis of variance
CNS: central nervous system
CSF: cerebrospinal fluid
FOME: Fuld object memory evaluation
LSD: least square difference
MMSE: Mini Mental Status Examination
NDDs: neurodegenerative disorders
PD: Parkinson’s disease
ROC: receiver operating characteristic
SE: sensitivity
SKT: short cognitive test
TBI: traumatic brain injury
TMT: trail making test
VaD: vascular dementia
